# Interactions among nutrition, metabolism and the immune system in the context of starvation and nutrition-stimulated obesity

**DOI:** 10.1038/s41387-025-00383-w

**Published:** 2025-06-10

**Authors:** Borros Arneth

**Affiliations:** 1 Institute of Laboratory Medicine and Pathobiochemistry, Molecular Diagnostics, Philipps University Marburg, Baldinger Str, Marburg, Germany; 2 Institute of Laboratory Medicine and Pathobiochemistry, Molecular Diagnostics, Justus Liebig University Giessen, Giessen, Germany

**Keywords:** Diabetes complications, Obesity

## Abstract

The endogenous intestinal microflora and environmental factors, such as diet, play central roles in immune homeostasis and reactivity. The microflora and diet both influence body weight and insulin resistance, notably through their effects on adipose cells. The aim of this study was to provide an update on how nutrient-derived factors (mostly focusing on fatty acids and glucose) impact the innate and acquired immune systems, including the immune system in the gut and its associated bacterial flora. The main source of fuel for energy-demanding immune cells is glucose. Insulin-responsive adipose tissue and Toll-like receptors (TLRs), which are part of the innate immune system and expressed in immune cells, intestinal cells, and adipocytes, are essential actors in the complex balance that ensures systemic immune and metabolic health. Leptin decreases during weight loss and increases brain activity in regions involved in the cognitive, emotional, and sensory control of food intake; restoring leptin levels maintains weight loss and reverses the alterations in brain activity. Obesity-triggering nutrients affect adipocytes, whereas proinflammatory leptin prompts the generation of cytokines and T cells. Collectively, data on nutrients demonstrate that starvation culminates in fat depletion, which then impacts the immune system. In people with obesity, inflammation originates largely from adipose tissue.

## Introduction

The continued existence of an organism is based on its ability to acquire and store nutrients for immediate and future metabolic processes. Multicellular organisms have developed strong barriers, including the skin and mucosa, to physically deter microorganisms from accessing tissues [1]. Moreover, dedicated immune cells are situated in every organ to identify and neutralize viruses or microbes that traverse such barriers. Nonetheless, a robust immune system comes at a high cost. Nutritional status substantially impacts the immune system, as undernutrition and overnutrition both influence immunological responses [2]. Starvation reduces the ability of the immune system to combat infection by depriving it of crucial nutrients required for cell activity. However, the increased nutrient intake in overnutrition, especially in people with obesity, can induce inflammation, which contributes to metabolic disorders such as insulin resistance [3]. Therefore, a dynamic equilibrium between the assimilation of nutrients and their utilization by the body is essential to sustain immune homeostasis and avoid activating autoimmune responses.

Certain immune cells, such as T-cell clones, may not be as important because they rarely meet their stimulating antigens [4]. The need for a proficient, extremely responsive immune system somewhat warrants the fact that likely redundant cells and low-efficiency energy-dispensing methods are preferred and have advantages when the immune system encounters a pathogen or antigen [5]. Although the immune system is not granted free rein to utilize calories, energy-proficient mechanisms are employed to meet the demand for energy under homeostatic conditions. Conversely, when nutrients are in low supply, the functionality of immune cells is severely impaired [6].

Nutrients are stored in adipose tissue, a critical endocrine organ that relays information concerning the size of the adipose tissue to the body. Such information controls a variety of functions, such as ingestion and immune cell function [7]. Although the pancreas measures the amount of glucose in the blood, adipose tissue functions as a sensor to determine nutrient availability [8], specifically by sensing to fat accumulation in adipocytes. The interaction between the immune system and adipose tissue becomes especially significant during periods of nutrient scarcity or overabundance. When fat stores are reduced during malnutrition, adipose tissue sends signals that suppress immune system activity, reducing energy consumption while preserving important bodily processes; this immune suppression allows the body to preserve energy by allocating resources for critical functions. In contrast, in the context of people with obesity, adipose tissue becomes overloaded with nutrients, resulting in chronic inflammation that affects normal metabolic function [9].

During starvation, for example, adipose tissue tends to shrink due to nutrient deficiency. In this case, adipocytes communicate with the body to minimize metabolic activity and optimize energy expenditure. Similarly, immune cell activity and populations are curtailed [10]. In contrast, when nutrients are plentiful, nutrient usage by the immune system is allowed to increase, but this can lead to overstimulation in the event of excessive feeding [11]. The availability of fast foods, characterized by high caloric content but low energy potential, has increased the number of people with obesity in recent decades. Consequently, cases of numerous obesity-related ailments, including type II diabetes and metabolic diseases, have increased. A correlation has been established between the immune system and the development of type II diabetes [12]. Type II diabetes is closely linked to the accumulation of proinflammatory macrophages in visceral adipose tissue (VAT) [13]. Evidently, adipose tissue plays a critical intermediary role with respect to cell activity and nutrient availability [14]. This paper highlights interactions among nutrition, metabolism and the immune system with reference to starvation and nutrient-stimulated obesity.

## Methods

### Search strategy

The search strategy focused on systematic reviews published between 2007 and 2017. Systematic reviews were used to assess and synthesize evidence regarding the interactions among nutrition, metabolism and the immune system. The reviews were collected from different online databases, including PubMed, CINAHL, MEDLINE, Science Direct, Elsevier, PMC, and Wiley Online Library. The search terms included nutrition, metabolism, systematic reviews, immune systems and nutritional elements. First, a total of 100 articles were reviewed. However, after the abstracts were read, 20 studies were excluded because they did not contain the search terms. Of the remaining 80 articles, 10 were excluded, and 70 were ultimately included. These 70 articles included both systematic reviews and peer-reviewed journal articles highlighting the effects of nutritional elements on the immune system and metabolism.

## Results

A summary of the most important mechanisms involved in nutrient metabolism and immune interactions, particularly in people with obesity and malnutrition, is provided in Table 1.

**Table 1:** Important Mechanisms.

Mechanism	What it Does	Impact on Health (Positive or Negative)	Citations
Leptin Resistance in Obesity	Leptin, the hormone that signals satiety, loses effectiveness in obesity. The brain becomes immune to the leptin signals to stop caloric intake, overriding the body’s natural appetite control. As eating persists unrestrained, fat stores amass, perpetuating the condition.	Negative: Overeating results in the storage of more fat; this leads to increased obesity. Hence, a negative cycle develops, that results in weight gain.	[77]
Adiponectin and Insulin Sensitivity	Adiponectin increases the body’s sensitivity to insulin and increases nutrient storage for fat in a healthy and positive manner. Adiponectin levels are high in lean persons and low in individuals with obesity.	Negative: Individuals with obesity have low levels of adiponectin and can develop insulin resistance; such individuals are also at high risk of developing type II diabetes and inflammation.	[35]
Pro-inflammatory Adipokines	Adipokines like leptin and resistin are hormones that promote inflammation. In people with obesity, increasing levels of proinflammatory adipokines contribute to adverse immune responses.	Downside: Excess pro-inflammatory adipokines lead to chronic inflammation, insulin resistance, and metabolic diseases.	[41]
White Adipose Tissue (WAT)	Leptin and adiponectin both signal the brain to increase metabolism and are released by WAT, which stores energy in fat cells. Visceral fat is a major concern as it directly leads to obesity due to excess WAT.	WAT deposits in other organs (visceral fat) lead to metabolic disorders like type II diabetes.	[15, 16, 26]
Brown Adipose Tissue (BAT)	BAT contributes to thermogenesis by combusting calories to produce heat, especially under cold conditions, and ameliorates glucose metabolism.	Positive: The activation of BAT may further increase insulin sensitivity and contribute to weight control by burning surplus fat.	[15, 18, 21–23]
Macrophages in Adipose Tissue (ATMs)	In lean individuals, macrophages in fat tissue perform tissue repair. In the case of people with obesity, these macrophages adopt a pro-inflammatory phenotype and mediate harmful immune responses.	Pro-inflammatory macrophages enhance chronic inflammation, insulin resistance, and disease vulnerability.	[46]
AMP-activated Protein Kinase (AMPK)	AMPK activates processes for procuring and expending energy amid energy scarcity while suppressing wasteful metabolism when reserves are replete.	Advantageous balanced energy signaling involves AMPK and curtails excessive weight and the metabolic dysfunction it fosters.	[51, 53, 54]
Gut Microbiota and Energy Extraction	The gut microbiota aids in digesting food and regulating the energy consumed through diet. In people with obesity, some bacteria stimulate higher energy absorption levels, thus promoting fat accumulation.	Negative: The increased calorie absorption due to an imbalance in gut microbiota and subsequent storage in fat depots promotes obesity.	[71, 74]
Starvation and Immune Function	While starving, the body reduces immune activity to reduce energy usage, predisposing it to infection.	Negative: Malnutrition weakens immunity and puts individuals, especially children, at risk of contracting diseases.	[3, 65–67]
Visceral Adipose Tissue (VAT) in Obesity	VAT wraps around internal organs and houses numerous immune cells; in people with obesity, VAT becomes inflamed as part of insulin resistance and metabolic dysfunction.	Negative: Chronic inflammation in VAT causes extreme health complications that include type II diabetes and cardiovascular diseases.	[50, 62, 64]

### Adipose tissue and metabolism

Adipose tissue is categorized as white adipose tissue (WAT), which stores energy, and brown adipose tissue (BAT), which provides nonshivering thermogenesis [15]. WAT, which contains adipocytes with droplets of unilocular lipids, stores energy for later use and balances energy levels in the body [16]. In contrast, BAT potentially gets its color from mitochondrial membranes [17]. While WAT primarily serves as an energy reservoir, BAT functions more actively in heat production, especially during cold exposure [18]. The ability of BAT to generate heat stems from its high mitochondrial density, allowing fatty acid oxidation and heat production instead of energy storage. The thermogenic role of BAT is particularly critical for newborns, who rely on BAT to maintain body temperature in cold environments [19]. While animals, especially mice, have considerable quantities of BAT, it is common in only newborn humans and some adults [20].

Recent studies have revealed that BAT can also play a role in combating obesity and metabolic disorders [21, 22]. BAT activation was demonstrated to improve glucose metabolism and insulin sensitivity in adults, suggesting that increasing BAT activity could be a therapeutic approach to prevent obesity-related disorders. Furthermore, the discovery of “beige” adipocytes—initially white cells that may adopt brown-like features under specific stimuli—has offered new paths for developing obesity treatments that activate beige fat cells to promote thermogenesis and energy expenditure [23]. WAT can be found in different organs of the body, but its distribution is determined by hormones [24]. Therefore, unique organs containing WAT are found in the abdominal cavity: VAT and subcutaneous fat [24]. WAT can also be found in the kidney [25]. Since it can store and balance energy in the body, WAT helps regulate metabolism by excreting cytokines and hormones, including adiponectin and leptin, which regulate metabolism and feeding [26]. Excreted adipokines can modulate the immune system. The accumulation of visceral fat is related to metabolic disorders such as type II diabetes, dyslipidemia, and insulin resistance [27].

Adipose organs have been widely investigated in mice, and the data demonstrate that fat tissues develop from several progenitors. Numerous brown fat reservoirs are generated from Myf5+Pax3+ progenitors; however, some BAT depots can develop without expression of these transcription factors [28]. On the other hand, numerous white adipose cells are obtained from Myf5-Pax3- progenitors, with specific exceptions. For example, anterior subcutaneous WAT development critically relies on Myf5+Pax3+ progenitors [29]. There are distinctions in WAT between female and male rodents: for example, perigonadal WAT is obtained from Pax3- progenitors in females, whereas the development of this tissue depends on Pax3 in males [30]. For all these reasons, it is important to consider fat development when investigating people with obesity.

An acceptable body weight has been clearly defined clinically. A body mass index (BMI) of 18.5 to 25 kg/m^2^ is viewed as normal, whereas individuals with a BMI below 18 kg/m^2^ are underweight, those with a BMI above 25 kg/m^2^ are overweight, and those with a BMI greater than 30 kg/m^2^ are people with obesity [31]. Biologically, the differences among people with obesity, lean, and malnourished individuals are inadequately defined. With excessive consumption, the effect on overall body weight is insignificant because of the ability of adipose cells to increase energy density [32]. Nonetheless, excessive nutrient consumption has a significant effect on adipocytes. In addition, adipocytes can transition from a proadipogenic state to an antiadipogenic state at the endocrine level. Adiponectin is generated by adipose tissue and acts as an insulin homolog by impairing gluconeogenesis in the liver and increasing glucose uptake and free fatty acid esterification [33]. Mice deficient in adiponectin become insulin resistant and accumulate lipids in skeletal muscle [34]. The levels of adiponectin are negatively associated with the volume adipose tissue [35]. Under lean conditions, adipocytes generate considerable amounts of adiponectin, promoting fat accumulation in adipose tissue [36]. In people with obesity, adipocytes decrease adiponectin production in response to the adipokine leptin. In turn, leptin sends satiety signals to the central nervous system that stimulate the effects of ghrelin, the hunger-triggering cytokine generated by the gut [37]. Leptin also hinders lipogenesis while increasing glucose metabolism and preventing glucose accumulation in adipocytes [38]. The exogenous administration of leptin prevents the progression of obesity resulting from overfeeding due to low leptin levels [39].

### Effects of adipokines on immune cells

Adipose tissue plays an important role in controlling metabolic processes and regulating immune cells. However, these processes rely on large amounts of adipokines. Fat has been considered an endocrine organ since the discovery of leptin, which led to the discovery of approximately fifty molecules produced by adipose tissue [40]. Adipokines are critical mediators of inflammation and metabolic balance [41]. For example, by encouraging T-cell activation, leptin contributes to enhancing immune responses and controlling appetite. However, adiponectin possesses anti-inflammatory qualities, and its levels are negatively correlated with obesity; slim people have higher quantities of adiponectin. Additionally, s adiponectin increases insulin sensitivity to protect against metabolic diseases.

Although adipokines potentially function in numerous ways, their impacts on the immune system can be grouped into two categories. The category that includes angiopoietin-like protein 2 (ANGPTL2), resistin, and leptin helps promote metabolic dysfunction and inflammation [42]. Leptin, in particular, links the nutritional state to immune function, as it is produced in greater amounts in individuals with obesity and contributes to chronic inflammation [43]. In contrast, the anti-inflammatory effects of adiponectin help counteract the negative impacts of leptin, making adiponectin a crucial factor in maintaining metabolic homeostasis in healthy individuals.

The other category of adipokines is anti-inflammatory and includes visfatin, adipsin, and adiponectin [44]. Changes in the levels of adipokines in these two categories are determined by the mass of adipose tissue and thus can be triggered by obesity. However, the actual functions of adipokines and nutrients in the immune system and metabolism are not clear.

### Immune mechanism in adipose tissue during homeostasis

The interface between cells and tissues that store nutrients and the immune system has been researched broadly in VAT [45]. Although VAT lacks a barricade function, it is populated with an enormous number of immune cells even under quiescent conditions. Regulatory immune cells within adipose tissue are essential for maintaining tissue homeostasis because they mediate the balance between pro- and anti-inflammatory responses. For example, adipose tissue-resident macrophages (ATMs) exist in both the lean and people with obesity. In lean individuals, ATMs predominantly exhibit an anti-inflammatory M2 phenotype, which supports tissue repair and insulin sensitivity. However, in abdominal fat-tissue of people with obesity, these macrophages shift toward a proinflammatory M1 phenotype, contributing to chronic inflammation and metabolic dysfunction [46].

The availability of molecules such as interleukin (IL)-10 and IL-1Ra endows these immune cells with the anti-inflammatory capabilities of adiponectin [47]. As mentioned above, adipocytes are the core immune cell subtypes in VAT that are responsible for cellular immunity [48]. Furthermore, several dedicated immune cell subtypes are involved in adipose tissue homeostasis. VAT includes CD4 T cells, which form the largest cluster of cells, the majority of which are FoxP3-expressing supervisory T cells. IL-10, generated from regulatory T cells (Tregs), is instrumental in preventing inflammation in VAT [49]. Nonetheless, the loss of Tregs culminates in acute inflammation in VAT while enhancing the evolution of insulin resistance as a result of nutrient-aggravated obesity [50]. Another vital cluster of cells is invariant-chain natural killer T cells, which are responsible for regulating immune cell activities during homeostasis.

At the molecular level, adenosine monophosphate (AMP)-activated protein kinase (AMPK) maintains energy homeostasis during starvation and fasting [51]. Cellular stress from hypoxia contributes to an increase in the AMP/ATP ratio. AMP stimulates AMPK to restore energy levels by triggering the catabolism of the considerable quantity of fatty acids, glycolysis, autophagy [52], and the oxidation of fatty acids [53]. AMPK also prevents anabolic pathways such as gluconeogenesis and the synthesis of fatty acids, glycogen, and triglycerides [54].

### Influence of nutrients on the immune system via adipose tissue

Good nutrition is useful in preventing malnutrition and noninfectious disorders. Because adipose tissue is the main nutrient storage area, nutrients significantly influence the phenotype of fat cells. High-fat and high-sugar diets promote inflammation in adipose tissue. Excess nutrients cause adipocyte hypertrophy and the infiltration of immune cells, especially macrophages. Obesity is associated with chronic low-grade inflammation, which is known to be a direct cause of type II diabetes and metabolic syndrome [55]. Owing to increased urbanization and lifestyle changes, the intake of energy, free sugar, salt, and fat is increasing [56]. Increased consumption of these nutrients is positively related to increases in adipose tissue, metabolic dysfunction, obesity, cellular oxidative stress, and other disorders [57].

However, nutrient-dense diets high in fiber, antioxidants, and polyunsaturated fats have anti-inflammatory effects on adipose tissue. These foods increase the production of adiponectin, an anti-inflammatory adipokine, while decreasing the infiltration of proinflammatory immune cells. As a result, proper nutrition is critical for modulating immunological responses within adipose tissue, which ultimately influences overall metabolic health.

Obesity-triggered changes in nutrient status are related to considerable increases in glucose and fatty acid levels. Diets rich in fatty acids inhibit the lipolytic role of adipocytes [58], whereas diets containing protein provide important amino acids for the synthesis of new proteins and the metabolism of lipids [59]. The composition and amount of protein in food affect metabolism in different ways [60]. In adipocytes, proteins prevent lipogenic enzymes, including glucose-6-phosphate dehydrogenase and fatty acid synthase [61].

### Immune response of VAT to obesity

Obesity has a strong effect on the immune system. In both mice and humans, type II diabetes triggered by obesity is correlated with reduced levels of inflammatory mediators [62]. Obesity leads to an increase in proinflammatory ATMs in VAT, which transition from the M1 phenotype to the M2 phenotype [63]. In overweight individuals and people with type II diabetes, M1 ATMs promote chronic inflammation, which is considered a risk factor for the progression of insulin resistance. Although the cause of the increased accumulation of immune cells in fat tissue in individuals with obesity is not known, it is believed that leptin increases the accumulation of adipose tissue. Obesity stimulates the progression of an inflammatory reaction that impacts all aspects of the immune system [64]. The ultimate immune response is the cause of chronic inflammation and the major factor that induces several disorders in the population with obesity.

### Starvation and the immune system

Starvation weakens the function of the immune system. Globally, starvation is one of the main health issues, as approximately 805 million individuals have insufficient food [65]. Unlike malnourished adults, most impacted children have a weakened immune system that increases their vulnerability to numerous diseases [66]. For this reason, malnutrition is the main cause of mortality in three million children annually. Both the innate and adaptive immune systems are suppressed by starvation. The production of key immune cells, such as T cells and neutrophils, is severely reduced by nutrient deficiency, leaving the body less well equipped to fight infection. In addition, malnourished individuals often experience decreased cytokine production and impaired macrophage and natural killer cell function, further compromising their immune defenses. Malnutrition can also cause thymic atrophy, which reduces the body’s ability to generate new T cells, thus weakening the overall immune response [67]. The physiological reaction to inadequate nutrients occurs in three stages.

During the fasting stage, the body is starved for several hours, and adipose lipolysis occurs to control fatty acid levels and empathic glycogenolysis to maintain blood glucose [68]. In the second stage, prolonged starvation occurs due to reduced glycogen stores. Afterward, the body switches to lipolysis to maintain energy; this process also produces glycerol and ketone bodies, the latter of which are used as fuel for organs [69]. This second phase lasts for several weeks, leading to significant weight loss. The third stage begins with considerable reductions in fat repositories and skeletal muscle to obtain amino acids for gluconeogenesis [70]. This state is associated with the loss of body mass and cannot be maintained for a long period. It is also characterized by decreased energy use, temperature, and immune function.

### Impact of nutrients on bacterial flora

The bacterial flora, also known as the microbiome or gut flora, depends on the human body as a place to live. The bacterial flora promotes human health to support its own survival, a process called symbiosis. The important bacteria that live in the gastrointestinal tract are necessary for human health. A study conducted in 2004 provided the first evidence that the bacterial flora may play a notable role in the energy balance and thus the development of obesity [71]. In this study, germ-free mice were colonized with bacteria from the intestines of normal mice. At fourteen days, the initially germ-free mice showed a noteworthy 60% increase in body fat content, despite decreased food intake. This finding highlights the ability of the microbiome to influence fat storage and energy balance, suggesting that gut bacteria could regulate the efficiency of calorie absorption from the diet. Some types of bacteria are better at extracting energy from food and could result in increased fat storage even when food consumption is decreased. Furthermore, changes in dietary habits, such as consuming meals high in fat or sugar, affect the composition of the gut microbiota, which may encourage the growth of bacteria that support energy storage and contribute to obesity [72].

More intriguing is the follow-up study in which germ-free mice were colonized by bacterial flora from mice with obesity [73]. Compared with the 27% fat increase in the mice colonized with bacteria from lean mice, the body fat of the germ-free mice colonized with bacteria from mice with obesity increased by 47%. This finding suggests that the bacterial flora may have unappreciated links to body fat. Further research has demonstrated that the composition of gut bacteria can influence not only fat storage but also overall metabolism. Certain bacteria, such as *Firmicutes*, are more prevalent in individuals with obesity and are thought to be more efficient at breaking down complex carbohydrates into short-chain fatty acids, which are then absorbed as calories. In contrast, bacteria such as *Bacteroidetes* are more common in lean individuals and may play a role in limiting fat storage by promoting faster energy expenditure [74].

To identify whether the bacterial flora can predict weight status, scientists have evaluated the fecal microbiota of infants and compared it with weight conditions later in life [75]. Children who became children with obesity had fewer bifidobacteria in their bacterial flora during infancy and childhood than did children who maintained a healthy weight. Regardless of whether this connection is causal, it is clear that obesity is linked to specific of the bacterial flora.

Recently, studies have aimed to describe the bacterial flora and characterize it on the basis of metabolic profiles, such as energy-harvesting capacity, instead of traditional taxonomy (genus, species, and phylum) [76]. A controversial subject is whether it is important to group bacterial flora by metabolic traits for the purpose of determining a therapeutic approach to obesity. However, it is not clear whether the impact of diet on bacterial flora, the connections among bacterial floras, and health outcomes will become more apparent with a new classification scheme.

### Obesity and leptin

Leptin is secreted by adipocytes and is thought to signal to the brain to prevent food intake and reduce weight [77]. This conclusion was reached based on the findings that rodents and humans lacking a functional leptin receptor or leptin itself show avid feeding and obesity. Thus, leptin treatment, especially leptin administration directly into the hypothalamus, is expected to significantly suppress dietary intake and lower fat stores and body weight in leptin-deficient animals [78]. Nevertheless, in most cases of people with obesity, leptin resistance occurs; in such cases, the individual has high circulating concentrations of leptin that are not sensed properly by the brain. Leptin resistance consequently disrupts the appropriate function of leptin, rending it unable to effectively regulate appetite or energy expenditure; hence, people with leptin resistance continue eating, resulting in obesity. There are three ways through which leptin resistance can develop: hypothalamic inflammation, high levels of free fatty acids, and problems related to leptin transport across the blood‒brain barrier. Thus, even in the presence of extremely high levels of leptin, its regulatory and downstream pathways do not tell the brain to reduce food intake and weight.

Nevertheless, the idea of leptin as an anti-obesity hormone has been questioned, as obesity is usually connected with high levels of leptin. In addition, humans and rodents who suffer from obesity due to a high-fat diet do not respond to leptin [79]. For example, in rodents, leptin is transported to the brain, binds to its receptor in the hypothalamus, and triggers JAK-STAT3 signaling, resulting in the destruction of orexigenic peptides and an increase in anorexigenic peptides. Although there is no clear dysfunction in leptin receptors in rodents with diet-induced obesity, transport of leptin across the blood‒brain barrier is decreased, and the level of SOCS3, an inhibitor of leptin signaling, is increased in the hypothalamus.

Leptin levels markedly decrease in response to fasting, suggesting substantial changes in energy balance and hormone levels [80]. Low leptin levels encourage overfeeding and the suppression of energy expenditure, immunity, and thyroid and reproductive hormone levels [81]. In rodents, low leptin levels increase orexigenic peptide levels and decrease anorexigenic peptide levels. Replenishment of leptin reverses these changes in hormone levels, metabolism, immunity, and hypothalamic neuropeptides. In addition, restoring leptin in patients without fat cells improves reproductive processes and reverses irregular glucose and lipid metabolism [82]. In an environment of plentiful food and limited exercise, this metabolic efficiency predisposes individuals toward obesity.

## Discussion

Adipose tissue is distributed throughout the body and can grow to house excess energy in the form of accumulated lipids, traits that differentiate adipose tissue from other tissues and organs in the body. Humans have two types of adipose tissue, i.e., WAT and BAT. There are two major depots of WAT—subcutaneous WAT and VAT around internal organs. The main physiological functions of WAT are insulation and energy storage. However, in individuals with obesity, excess VAT is closely related to metabolic disorders, such as type II diabetes and insulin resistance.

Obesity has a strong effect on the immune system. In both mice and humans, type II diabetes triggered by obesity is correlated with reduced levels of inflammatory mediators. To understand the role of macrophages in adipose tissue, identifying the difference between subcutaneous fat and visceral fat is important. Visceral fat, such as perinephric, omentum, and mesenteric adipose tissue, is connected to internal organs. Obesity stimulates the progression of an inflammatory reaction that integrates all aspects of the immune system.

In addition, leptin has a similar structure to that of IL-6, and both proteins signal through STA3 [83]. Obesity-triggering nutrients affect adipocytes. The proinflammatory hormone leptin triggers the generation of cytokines and T cells [84]. Similarly, resistin leads to diabetes, especially when increases in its levels are associated with insulin resistance [85]. Although there is a strong relationship between insulin resistance and resistin, the findings are conflicting in human and mouse studies [86]. However, resistin can promote inflammatory reactions by activating IL-6 secretion. Leptin is secreted by adipocytes and is thought to signal to the brain to prevent food intake and reduce weight.

Adipsin is an anti-inflammatory adipokine that preserves the normal function of pancreatic cells. Mice without adipsin are vulnerable to impaired glucose tolerance due to decreased insulin production and insulinopenia. In general, anti-inflammatory and proinflammatory adipokines act as signals between the immune system and adipose tissue that are produced on the basis of the nutritional state of the organism. Metabolism is important for organism's survival. Adipose tissue is the primary organ for storing nutrients and regulating metabolism within the immune system and other organs. However, adipose tissue is only one of the elements that guarantee the wellness of organisms.

## Conclusion

Nutrition research has shown that starvation leads to fat depletion, which in turn affects the immune system. In the context of people with obesity, the main cause of inflammation is adipose tissue. Adipokines play different roles in the immune system. For example, dysfunction of the peptide hormone leptin is responsible for obesity. In contrast, adiponectin has anti-inflammatory features and likely affects macrophage and monocyte activity. Nonetheless, as individuals with obesity experience chronic inflammation as a result of immune system stimulation, they are more vulnerable to comorbidities than individuals at a healthy weight. The increased rate of inflammatory diseases among individuals with obesity demonstrates the suppressed reactions of their immune systems to pathogens. Mutation of the leptin gene also leads to diabetes, as leptin is crucial in mediating energy metabolism. Therefore, leptin is essential to the immune system and to the metabolism of lipids and glucose. In addition to leptin, adipokines such as ANGPTL2 and resistin have inflammatory properties. Therefore, obesity is related to an increased level of leptin, which supports inflammation and activates Th1 cells and the production of proinflammatory cytokines.

## References

[1] Wensveen FM, Gisbergen KP, Eldering E. The fourth dimension in immunological space: How the struggle for nutrients selects high-affinity lymphocytes. Immunol Rev. 2012;249:84–103. 22889217 10.1111/j.1600-065X.2012.01156.x

[2] Morales F, Montserrat-de la Paz S, Leon MJ, Rivero-Pino F. Effects of malnutrition on the immune system and infection and the role of nutritional strategies regarding improvements in children’s health status: a literature review. Nutrients. 2023;16:1. 38201831 10.3390/nu16010001 PMC10780435

[3] Wondmkun YT. Obesity, insulin resistance, and type 2 diabetes: associations and therapeutic implications. Diabetes Metab Syndrome Obes. 2020;13:3611–6. 10.2147/DMSO.S275898 PMC7553667 33116712

[4] Gubser PM, Bantug GR, Razik L, Fischer M, Dimeloe S, Hoenger G, et al. Rapid effector function of memory CD8+ T cells requires an immediate-early glycolytic switch. Nat Immunol. 2013;14:1064. 23955661 10.1038/ni.2687

[5] van der Windt GJ, Everts B, Chang CH, Curtis JD, Freitas TC, Amiel E, et al. Mitochondrial respiratory capacity is a critical regulator of CD8+ T cell memory development. Immunity. 2012;36:68–78. 22206904 10.1016/j.immuni.2011.12.007 PMC3269311

[6] Stevens GA, Singh GM, Lu Y, Danaei G, Lin JK, Finucane MM, et al. National, regional, and global trends in adult overweight and obesity prevalences. Popul Health Metr. 2012;10:22. 23167948 10.1186/1478-7954-10-22 PMC3543235

[7] Moschen AR, Kaser A, Enrich B, Mosheimer B, Theurl M, Niederegger H, et al. Visfatin, an adipocytokine with proinflammatory and immunomodulating properties. J Immunol. 2007;178:1748–58. 17237424 10.4049/jimmunol.178.3.1748

[8] Lidell ME, Betz MJ, Leinhard OD, Heglind M, Elander L, Slawik M, et al. Evidence for twotypes of brown adipose tissue in humans. Nat Med. 2013;19:631. 23603813 10.1038/nm.3017

[9] Chait A, Den Hartigh LJ. Adipose tissue distribution, inflammation and its metabolic consequences, including diabetes and cardiovascular disease. Front Cardiovasc Med. 2020;7:522637. 10.3389/fcvm.2020.00022 PMC7052117 32158768

[10] Ouchi N, Ohashi K, Shibata R, Murohara T. Adipocytokines and obesity-linked disorders. Nagoya. J Med Sci. 2012;74:19. PMC4831247 22515108

[11] Fuster JJ, Ouchi N, Gokce N, Walsh K. Obesity-induced changes in adipose tissue microenvironment and their impact on cardiovascular disease. Circ Res. 2016;118:1786–807. 27230642 10.1161/CIRCRESAHA.115.306885 PMC4887147

[12] Aprahamian TR, Sam F. Adiponectin in cardiovascular inflammation and obesity. Int J Inflamm. 2011;2011:1–11. 10.4061/2011/376909 PMC3175407 21941676

[13] Kern PA, Saghizadeh M, Ong JM, Bosch RJ, Deem R, Simsolo RB. The expression of tumor necrosis factor in human adipose tissue. Regulation by obesity, weight loss, and relationship to lipoprotein lipase. J Clin Investig. 1995;95:2111–9. 7738178 10.1172/JCI117899 PMC295809

[14] Odegaard JI, Chawla A. The immune system as a sensor of the metabolic state. Immunity. 2013;38:644–54. 23601683 10.1016/j.immuni.2013.04.001 PMC3663597

[15] Azevedo FR, Brito BC. Influence of nutritional variables and obesity on health and metabolism. Rev da Assoc ção Méd Brasileira. 2012;58:714–23. 10.1590/s0104-42302012000600018 23250102

[16] Saxena A, Chumanevich A, Fletcher E, Larsen B, Lattwein K, Kaur K, et al. Adiponectin deficiency: role in chronic inflammation induced colon cancer. Biochim Biophys Acta (BBA)-Mol Basis Dis. 2012;1822:527–36. 10.1016/j.bbadis.2011.12.006 PMC3961716 22198319

[17] Tilg H, Moschen AR. Role of adiponectin and PBEF/visfatin as regulators of inflammation: involvement in obesity-associated diseases. Clin Sci. 2008;114:275–88. 10.1042/CS20070196 18194136

[18] Lu X, Solmonson A, Lodi A, Nowinski SM, Sentandreu E, Riley CL, et al. The early metabolomic response of adipose tissue during acute cold exposure in mice. Sci Rep. 2017;7:3455. 28615704 10.1038/s41598-017-03108-x PMC5471228

[19] Bienboire-Frosini C, Wang D, Marcet-Rius M, Villanueva-García D, Gazzano A, Domínguez-Oliva A, et al. The role of brown adipose tissue and energy metabolism in mammalian thermoregulation during the perinatal period. Animals. 2023;13:2173. 37443971 10.3390/ani13132173 PMC10339909

[20] Wilk S, Scheibenbogen C, Bauer S, Jenke A, Rother M, Guerreiro M, et al. Adiponectin is a negative regulator of antigen-activated T cells. Eur J Immunol. 2011;41:2323–32. 21538348 10.1002/eji.201041349

[21] Liu X, Zhang Z, Song Y, Xie H, Dong M. An update on brown adipose tissue and obesity intervention: Function, regulation and therapeutic implications. Front Endocrinol. 2023;13:1065263. 10.3389/fendo.2022.1065263 PMC9874101 36714578

[22] Dodangeh M, Dodangeh M. Metabolic regulation and the anti-obesity perspectives of brown adipose tissue (BAT); a systematic review. Obes Med. 2020;17:100163.

[23] Magro BS, Dias DP. Brown and beige adipose tissue: new therapeutic targets for metabolic disorders. Health Sci Rev. 2024;25:100148.

[24] Tsang JY, Li D, Ho D, Peng J, Xu A, Lamb J, et al. Novel immunomodulatory effects of adiponectin on dendritic cell functions. Int Immunopharmacol. 2011;11:604–9. 21094289 10.1016/j.intimp.2010.11.009

[25] Sanchez-Gurmaches J, Guertin DA. Adipocyte lineages: tracing back the origins of fat. Biochim Biophys Acta (BBA)-Mol Basis Dis. 2014;1842:340–51. 10.1016/j.bbadis.2013.05.027 PMC3805734 23747579

[26] Betz MJ, Enerbäck S. Human brown adipose tissue: what we have learned so far. Diabetes. 2015;64:2352–60. 26050667 10.2337/db15-0146

[27] Leibel RL. Molecular physiology of weight regulation in mice and humans. Int J Obes. 2009;32:S98. 10.1038/ijo.2008.245 PMC2682360 19136999

[28] Nedergaard J, Bengtsson T, Cannon B. Unexpected evidence for active brown adipose tissue in adult humans. Am J Physiol Endocrinol Metab. 2007;293:E444–52.347. 17473055 10.1152/ajpendo.00691.2006

[29] Waldén TB, Hansen IR, Timmons JA, Cannon B, Nedergaard J. Recruited vs. nonrecruited molecular signatures of brown, “brite,” and white adipose tissues. Am J Physiol Endocrinol Metab. 2011;302:E19–31. 21828341 10.1152/ajpendo.00249.2011

[30] Sethi JK, Vidal-Puig AJ. Thematic review series: adipocyte biology. Adipose tissue function and plasticity orchestrate nutritional adaptation. J lipid Res. 2007;48:1253–62. 17374880 10.1194/jlr.R700005-JLR200 PMC4303760

[31] Lee YH, Petkova AP, Konkar AA, Granneman JG. Cellular origins of cold-induced brown adipocytes in adult mice. FASEB J. 2014;29:286–99. 25392270 10.1096/fj.14-263038 PMC4285542

[32] Wensveen FM, Valentić S, Šestan M, Wensveen TT, Polić B. Interactions between adipose tissue and the immune system in health and malnutrition. InSeminars Immunol. 2015;25:322–33. 10.1016/j.smim.2015.10.006 26603491

[33] Ouchi N, Parker JL, Lugus JJ, Walsh K. Adipokines in inflammation and metabolic disease. 359 Nat Rev Immunol. 2011;11:85. 21252989 10.1038/nri2921 PMC3518031

[34] Cristancho AG, Lazar MA. Forming functional fat: a growing understanding of adipocyte differentiation. Nat Rev Mol cell Biol. 2011;12:722. 21952300 10.1038/nrm3198 PMC7171550

[35] Cinti S. The adipose organ at a glance. Dis Models Mech. 2012;5:588–363 94. 10.1242/dmm.009662 PMC3424455 22915020

[36] Frontini A, Cinti S. Distribution and development of brown adipocytes in the murine and human adipose organ. Cell Metab. 2010;11:253–6. 20374956 10.1016/j.cmet.2010.03.004

[37] Cinti S. Between brown and white: novel aspects of adipocyte differentiation. Ann Med. 2011;43:104–15. 21254898 10.3109/07853890.2010.535557

[38] Nedergaard J, Bengtsson T, Cannon B. Three years with adult human brown adipose tissue. Ann N Y Acad Sci. 2010;1212. 10.1111/j.1749-6632.2010.05905.x 21375707

[39] Blüher M, Mantzoros CS. From leptin to other adipokines in health and disease: facts and expectations at the beginning of the 21st century. Metab Clin Exp. 2015;64:131–45. 25497344 10.1016/j.metabol.2014.10.016

[40] Nakamura K, Fuster JJ, Walsh K. Adipokines: a link between obesity and cardiovascular disease. J Cardiol. 2014;63:250–9. 24355497 10.1016/j.jjcc.2013.11.006 PMC3989503

[41] Kirichenko TV, Markina YV, Bogatyreva AI, Tolstik TV, Varaeva YR, Starodubova AV. The role of adipokines in inflammatory mechanisms of obesity. Int J Mol Sci. 2022;23:14982. 36499312 10.3390/ijms232314982 PMC9740598

[42] Toussirot E, Streit G, Wendling D. The contribution of adipose tissue and adipokines to inflammation in joint diseases. Curr Med Chem. 2007;14:1095–100. 17456023 10.2174/092986707780362826

[43] Pérez-Pérez A, Sánchez-Jiménez F, Vilariño-García T, Sánchez-Margalet V. Role of leptin in inflammation and vice versa. Int J Mol Sci. 2020;21:5887. 32824322 10.3390/ijms21165887 PMC7460646

[44] Malik VS, Willett WC, Hu FB. Global obesity: Trends, risk factors and policy implications. Nat Rev Endocrinol. 2013;9:13. 23165161 10.1038/nrendo.2012.199

[45] Ferreira AV, Menezes-Garcia Z, Viana JB, Mário ÉG, Botion LM. Distinct metabolic pathways trigger adipocyte fat accumulation induced by high-carbohydrate and high-fat diets. Nutrition. 2014;30:1138–43. 24976420 10.1016/j.nut.2014.02.017

[46] Yao J, Wu D, Qiu Y. Adipose tissue macrophage in obesity-associated metabolic diseases. Front Immunol. 2022;13:977485. 36119080 10.3389/fimmu.2022.977485 PMC9478335

[47] Odegaard JI, Chawla A. Pleiotropic actions of insulin resistance and inflammation in metabolic homeostasis. Science. 2013;339:172–7. 23307735 10.1126/science.1230721 PMC3725457

[48] Neuschwander Tetri BA. Hepatic lipotoxicity and the pathogenesis of nonalcoholic steatohepatitis: The central role of nontriglyceride fatty acid metabolites. Hepatology. 2010;52:774–88. 20683968 10.1002/hep.23719

[49] Hotamisligil GS, Erbay E. Nutrient sensing and inflammation in metabolic diseases. Nat Rev Immunol. 2008;8:923. 19029988 10.1038/nri2449 PMC2814543

[50] El Khoury D, Anderson GH. Recent advances in dietary proteins and lipid metabolism. Curr Opin Lipido. 2013;24:207–13. 10.1097/MOL.0b013e3283613bb7 23619369

[51] Pradelli LA, Beneteau M, Chauvin C, Jacquin MA, Marchetti S, Munoz-Pinedo C, et al. Glycolysis inhibition sensitizes tumor cells to death receptors-induced apoptosis by AMP kinase activation leading to Mcl-1 block in translation. Oncogene. 2010;29:1641. 19966861 10.1038/onc.2009.448

[52] Mongini PK, Inman JK, Han H, Fattah RJ, Abramson SB, Attur M. APRIL and BAFF promote increased viability of replicating human B2 cells via mechanism involving cyclooxygenase 2. J Immunol. 2006;176:6736–51. 16709833 10.4049/jimmunol.176.11.6736

[53] Pereira JP, Kelly LM, Cyster JG. Finding the right niche: B-cell migration in the early phases of T-dependent antibody responses. Int Immunol. 2010;22:413–9. 20508253 10.1093/intimm/dxq047 PMC2877811

[54] Bannard O, Kraman M, Fearon DT. Secondary replicative function of CD8+ T cells that had developed an effector phenotype. Science. 2009;323:505–9. 19164749 10.1126/science.1166831 PMC2653633

[55] Zatterale F, Longo M, Naderi J, Raciti GA, Desiderio A, Miele C, et al. Chronic adipose tissue inflammation linking obesity to insulin resistance and type 2 diabetes. Front Physiol. 2020;10:1607. 32063863 10.3389/fphys.2019.01607 PMC7000657

[56] Flint HJ, Duncan SH, Scott KP, Louis P. Links between diet, gut microbiota composition and gut metabolism. Proc Nutr Soc. 2015;74:13–22. 25268552 10.1017/S0029665114001463

[57] Salcido J, Engel T, Cano K, Angulo P, Brookes R, Kepler H, et al. Effects of branch chain amino acid supplementation on energy intake in healthy weight and overweight/obese individuals. Stud J Sci Math. 2017:25.

[58] Illesca PG, Álvarez SM, Selenscig DA, Ferreira MD, Giménez MS, Lombardo YB, et al. Dietary soy protein improves adipose tissue dysfunction by modulating parameters related with oxidative stress in dyslipidemic insulin-resistant rats. Biomed Pharmacother. 2017;88:1008–15. 28178612 10.1016/j.biopha.2017.01.153

[59] Johnson AR, Makowski L. Nutrition and metabolic correlates of obesity and inflammation: clinical considerations–3. J Nutr. 2015;145:1131S–6S. 25833891 10.3945/jn.114.200758 PMC4410497

[60] Sun K, Kusminski CM, Scherer PE. Adipose tissue remodeling and obesity. J Clin Investig. 2011;121:2094–101. 21633177 10.1172/JCI45887 PMC3104761

[61] Galic S, Oakhill JS, Steinberg GR. Adipose tissue as an endocrine organ. Mol Cell Endocrinol. 2010;316:129–39. 19723556 10.1016/j.mce.2009.08.018

[62] Audain KA, Zotor FB, Amuna P, Ellahi B. Food supplementation among HIV-infected adults in Sub-Saharan Africa: impact on treatment adherence and weight gain. Proc Nutr Soc. 2015;74:517–25. 25761769 10.1017/S0029665115000063

[63] Hickman D, Jones MK, Zhu S, Kirkpatrick E, Ostrov DA, Wang X, et al. The effect of malnutrition on norovirus infection. MBio. 2014;5:e01032–13. 24595373 10.1128/mBio.01032-13 PMC3958801

[64] Danaei G, Andrews KG, Sudfeld CR, Fink G, McCoy DC, Peet E, et al. Risk factors for childhood stunting in 137 developing countries: a comparative risk assessment analysis at global, regional, and country levels. PLoS Med. 2016;13:e1002164. 27802277 10.1371/journal.pmed.1002164 PMC5089547

[65] Ibrahim MK, Zambruni M, Melby CL, Melby PC. Impact of childhood malnutrition on host defense and infection. Clin Microbiol Rev. 2017;30:919–71. 28768707 10.1128/CMR.00119-16 PMC5608884

[66] Lobstein T, Jackson-Leach R, Moodie ML, Hall KD, Gortmaker SL, Swinburn BA, et al. Child and adolescent obesity: part of a bigger picture. Lancet. 2015;385:2510–20. 25703114 10.1016/S0140-6736(14)61746-3 PMC4594797

[67] França TG, Ishikawa LL, Zorzella-Pezavento SF, Chiuso-Minicucci F, da Cunha MD, Sartori A. Impact of malnutrition on immunity and infection. J Venom Anim Toxins including Tropic Dis. 2009;15:374–90.

[68] Xu L, Kitade H, Ni Y, Ota T. Roles of chemokines and chemokine receptors in obesity-associated insulin resistance and nonalcoholic fatty liver disease. Biomolecules. 2015;5:1563–79. 26197341 10.3390/biom5031563 PMC4598764

[69] Puchalska P, Crawford PA. Multi-dimensional roles of ketone bodies in fuel metabolism, signaling, and therapeutics. Cell Metab. 2017;25:262–84. 28178565 10.1016/j.cmet.2016.12.022 PMC5313038

[70] Yazdi FT, Clee SM, Meyre D. Obesity genetics in mouse and human: back and forth, and back again. PeerJ. 2015;3:e856. 25825681 10.7717/peerj.856 PMC4375971

[71] Bäckhed F, Ding H, Wang T, Hooper LV, Koh GY, Nagy A, et al. The gut microbiota as an environmental factor that regulates fat storage. Proc Natl Acad Sci USA. 2004;101:15718–23. 15505215 10.1073/pnas.0407076101 PMC524219

[72] Cuevas-Sierra A, Ramos-Lopez O, Riezu-Boj JI, Milagro FI, Martinez JA. Diet, gut microbiota, and obesity: links with host genetics and epigenetics and potential applications. Adv Nutr. 2019;10:S17–30. 30721960 10.1093/advances/nmy078 PMC6363528

[73] Turnbaugh PJ, Ley RE, Mahowald MA, Magrini V, Mardis ER, Gordon JI. An obesity-associated gut microbiome with increased capacity for energy harvest. Nature. 2006;444:1027–31. 17183312 10.1038/nature05414

[74] Houtman TA, Eckermann HA, Smidt H, de Weerth C. Gut microbiota and BMI throughout childhood: the role of firmicutes, bacteroidetes, and short-chain fatty acid producers. Sci Rep. 2022;12:3140. 35210542 10.1038/s41598-022-07176-6 PMC8873392

[75] Kalliomaki M, Collado MC, Salminen S, Isolauri E. Early differences in fecal microbiota composition in children may predict overweight. Am J Clin Nutr. 2008;87:534–8. 18326589 10.1093/ajcn/87.3.534

[76] Arumugam M, Raes J, Pelletier E, Le Paslier D, Yamada T, Mende DR, et al. Enterotypes of the human gut microbiome. Nature. 2011;473:174–80. 21508958 10.1038/nature09944 PMC3728647

[77] Zhang Y, Proenca R, Maffei M, Barone M, Leopold L, Friedman JM, et al. Positional cloning of the mouse obese gene and its human homologue. Nature. 1994;372:425. 7984236 10.1038/372425a0

[78] Morton GJ, Cummings DE, Baskin DG, Barsh GS, Schwartz MW. Central nervous system control of food intake and body weight. Nature. 2006;443:289. 16988703 10.1038/nature05026

[79] Heymsfield SB, Greenberg AS, Fujioka K, Dixon RM, Kushner R, Hunt T, et al. Recombinant leptin for weight loss in obese and lean adults: a randomized, controlled, dose-escalation trial. Jama. 1999;282:1568–75. 10546697 10.1001/jama.282.16.1568

[80] Ahima RS, Prabakaran D, Mantzoros C, Qu D, Lowell B, Maratos-Flier E, et al. Role of leptin in the neuroendocrine response to fasting. Nature. 1996;382:250. 8717038 10.1038/382250a0

[81] Farooqi IS, Matarese G, Lord GM, Keogh JM, Lawrence E, Agwu C, et al. Beneficial effects of leptin on obesity, T cell hyporesponsiveness, and neuroendocrine/metabolic dysfunction of human congenital leptin deficiency. J Clin Investig. 2002;110:1093–103. 12393845 10.1172/JCI15693 PMC150795

[82] Rosenbaum M, Murphy EM, Heymsfield SB, Matthews DE, Leibel RL. Low dose leptin administration reverses effects of sustained weight-reduction on energy expenditure and circulating concentrations of thyroid hormones. J Clin Endocrinol Metab. 2002;87:2391–4. 11994393 10.1210/jcem.87.5.8628

[83] Cole SW, Nagaraja AS, Lutgendorf SK, Green PA, Sood AK. Sympathetic nervous system regulation of the tumour microenvironment. Nat Rev Cancer. 2015;15:563. 26299593 10.1038/nrc3978 PMC4828959

[84] Candia P, De Rosa V, Gigantino V, Botti G, Ceriello A, Matarese G. Immunometabolism of human autoimmune diseases: from metabolites to extracellular vesicles. FEBS Lett. 2017;591:3119–34. 10.1002/1873-3468.12733 28649760

[85] Elekofehinti OO, Ejelonu OC, Kamdem JP, Akinlosotu OB, Adanlawo IG. Saponins as adipokines modulator: a possible therapeutic intervention for type 2 diabetes. World J Diab. 2017;8:337. 10.4239/wjd.v8.i7.337 PMC5507830 28751956

[86] Rani V, Deep G, Singh RK, Palle K, Yadav UC. Oxidative stress and metabolic disorders: pathogenesis and therapeutic strategies. Life Sci. 2016;148:183–93. 26851532 10.1016/j.lfs.2016.02.002

